# The frequency and clinical significance of DNA polymerase beta (POLβ) expression in breast ductal carcinoma in situ (DCIS)

**DOI:** 10.1007/s10549-021-06357-7

**Published:** 2021-08-18

**Authors:** Abdulbaqi Al-Kawaz, Reem Ali, Michael S. Toss, Islam M. Miligy, Omar J. Mohammed, Andrew R. Green, Srinivasan Madhusudan, Emad A. Rakha

**Affiliations:** 1 Division of Cancer and Stem Cells, School of Medicine, The University of Nottingham, Nottingham, UK; 2 Department of Pathology, College of Dentistry, Al Mustansiriya University, Baghdad, Iraq; 3 Department of Pathology, Faculty of Medicine, Menoufia University, Menoufia, Egypt; 4 Department of Histopathology, Nottingham University Hospital NHS Trust, City Hospital Campus, Hucknall Road, Nottingham, NG5 1PB UK

**Keywords:** DCIS, Breast cancer, POLβ, DNA damage response, Prognosis

## Abstract

**Background:**

The prediction of clinical behaviour of breast ductal carcinoma in situ (DCIS) and its progression to invasive disease remains a challenge. Alterations of DNA damage repair mechanisms are associated with invasive breast cancer (BC). This study aims to assess the role of base excision repair (BER) DNA Polymerase Beta (POLβ) in DCIS.

**Methods:**

A cohort of DCIS comprising pure DCIS (*n* = 776) and DCIS coexisting with invasive BC (*n* = 239) were prepared as tissue microarrays. POLβ protein expression was assessed using immunohistochemistry and correlated with clinicopathological parameters and patient outcome. Preclinically, we investigated the impact of POLβ depletion on stem cell markers in representative DCIS cell line models.

**Results:**

Reduced POLβ expression was associated with aggressive DCIS features including high nuclear grade, comedo necrosis, larger tumour size, hormonal receptor negativity, HER2 overexpression and high Ki67 index. Combined low nuclear/low cytoplasmic POLβ expression showed the strongest association with the features’ characteristics of aggressive behaviour. There was a gradual reduction in the POLβ expression from normal breast tissue, to DCIS, with the lowest expression observed in the invasive BC. Low POLβ expression was an independent predictor of recurrence in DCIS patients treated with breast conserving surgery (BCS). *POLβ* knockdown was associated with a significant increase in cell stemness markers including SOX2, NANOG and OCT4 levels in MCF10-DCIS cell lines.

**Conclusion:**

Loss of POLβ in DCIS is associated with aggressive behaviour and it can predict recurrence. POLβ expression in DCIS provides an additional feature for patients’ risk stratification for personalised therapy.

**Supplementary Information:**

The online version contains supplementary material available at 10.1007/s10549-021-06357-7.

## Introduction

Breast cancer (BC) carcinogenesis is a multistep process that involves genetic and epigenetic changes which leads to gradually develops invasive breast cancer (IBC). This multistep process transforms normal ductal cells pre-invasive lesions and eventually into invasive disease [1]. In breast ductal carcinoma in situ (DCIS) the malignant epithelial cells are morphologically and genetically similar to their invasive BC counterparts, but they are confined within the mammary duct system. Prediction of DCIS progression to invasive disease or recurrence after initial excision remains a challenge [2].

The maintenance of genomic integrity is achieved by DNA protection from damage that could be induced by endogenous or exogenous factors [3]. DNA damage repair (DDR) is a complex mechanism and depends on the interaction between the various pathways [4]. Carcinogenesis is driven by impaired DNA repair [5–7]. DNA polymerase β (POLβ) is one of the DNA polymerase groups that appear to have paramount significance in genome integrity preservation [8–10]. Bases that have been damaged by oxidation, alkylation or ring saturation must be removed accurately by the base excision repair (BER) pathway [11]. POLβ is recruited to the impaired cells by cooperating with the BER scaffold protein (XRCC1) and the DNA damage repair protein (PARP1) [12–14]. POLβ is localised on chromosome 8p11 and it is a hot spot for chromosomal deletion and alterations associated with different types of cancers including BC [15–19]. Mutation in POLβ raises the mutation frequency which promotes carcinogenesis [20–22].

In this study, we hypothesised that POLβ provides prognostic and predictive value in DCIS. We utilised a large well-characterised cohort of DCIS to assess the clinical and molecular significance of POLβ expression in DCIS and determine its association with the disease progression.

## Materials and methods

### Study cohort

This retrospective study was carried out on a successive series of 1015 DCIS cases comprising pure DCIS (*n* = 776) and DCIS with synchronous invasive BC (DCIS mixed; *n* = 239) diagnosed and treated at the Breast Institute, City Hospital, Nottingham, United Kingdom. The demographic and histopathological data including age at diagnosis, mode of disease presentation (symptomatic or screen-detected), DCIS size, nuclear grade, the presence of necrosis and postoperative radiotherapy (RT), were collected. Molecular classification based on the expression of hormonal receptors [oestrogen and progesterone receptor (ER&PR)], HER2 status and Ki-67 proliferation index were available as previously described [23]. ER&PR positivity was defined when the positive nuclei of tumour cells were ≥ 1% [24]. Herceptin test method was used to assess HER2 where IHC score of 0 or 1 considered as negative, 2 + considered as equivocal and 3 + considered as positive [25]. Moreover, the Ki-67 proliferation index was defined as high if its nuclear expression in malignant epithelial cells was more than 14% [23] (Supplementary Table S1). Local recurrence-free interval (LRFI) was defined as the time between the primary surgical excision to the time of development ipsilateral recurrence as DCIS or invasive BC. Cases with contralateral breast cancer were censored at the time of development of the contralateral event.

### Analysis of POLβ mRNA in invasive BC

Due to the limited availability of transcriptomic data of the DCIS, the Molecular Taxonomy of Breast Cancer International Consortium (METABRIC) (*n* = 1980) data were used to validate the clinical and prognostic significance of POLβ in invasive BC [26].

### Evaluation of POLβ protein expression

**Fig. 1:**
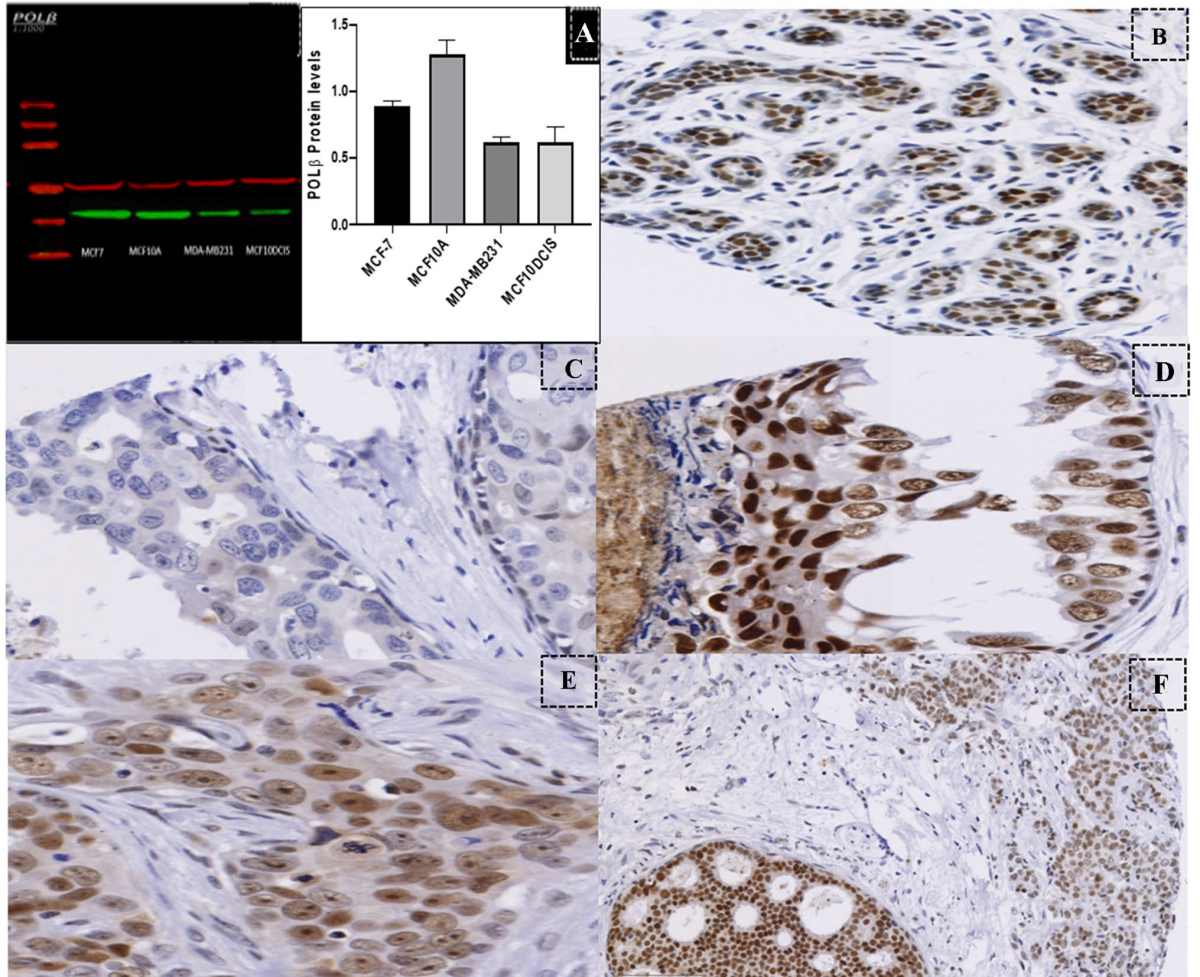
POL-β Western blot and IHC protein expression. **A** Western blot for POL-β antibody showed a single band at the predicted molecular weight 38 kDa in the cell lysates MCF7, MCF10A, MDA-MB231 and MCF10-DCIS, respectively (green bands). Tubulin used as an internal loading control and shows a single band as a standard control (red band) at the predicted molecular weight 55 kDa in all cell lysates. W.B chart was presented the higher level of POL-β protein was expressed in MCF10A, MCF7, MCF10DCIS, and MDA-MB 231, respectively. **B**–**F** POL-β protein expression in DCIS. **B** Normal terminal ductal lobular units, lined by single layer of epithelial cells (X10). **C** Negative IHC expression (X20). **D** Strong IHC expression in DCIS (X20). **E** Weak to moderate IHC expression in IBC component (X20). **F** Mixed DCIS coexistent with IBC (X10)

Western blotting was performed to validate the anti-POLβ antibody specificity (Abcam; rabbit polyclonal ab26343. Lot No. GR 284,224–3). A panel of human cell lysates was used including MCF10DCIS, MCF7, MCF10A and MDA-MB-231. They were purchased from the American Type Culture Collection (Rockville, MD, USA). A single specific band at the predicted size of 38 kDa was achieved using an antibody dilution at 1:1000 and incubated overnight at 4 °C. Anti-beta tubulin (mouse monoclonal anti-beta tubulin antibody Abcam) 55 kDa was included in the Western Blot as a loading control (Fig. 1A).

The assessment of the expression of POLβ protein in DCIS by immunohistochemistry (IHC) was conducted on 4 µm tissue microarray sections (TMA) and full-face tissue Sects. (10 cases) of formalin-fixed paraffin-embedded (FFPE) blocks using the Novocastra Novolink polymer detection system (Leica, Newcastle, UK) following the manufacturer’s guidelines. Subsequently, samples were incubated for 30 min at room temperature with the POLβ primary antibody optimally diluted in 1:500 in the Leica antibody diluent (Supplementary material and method S1, S2).

The scoring, based on a semi-quantitative histochemical assessment scoring method (*H*-Score), was conducted on POLβ nuclear and cytoplasmic expression. H-score took into consideration the staining intensity (negative, weak, moderate and strong expression as 0, 1, 2 and 3, respectively) and the percentage of the stained tumour cells. The result of scoring was gained by multiplying the intensity of staining by the percentage of expression in the nucleus and cytoplasm of the tumour cells. The score was expressed in a range of 0–300 [27]. All cores with less than 15% tumour were excluded from scoring. DCIS component and invasive component in DCIS mixed with invasive were scored separately. Moreover, whenever present in the tissue cores, expression of POLβ within the adjacent normal terminal ductal lobular unit (TDLUs) was also assessed (*n* = 50). X-tile (X-tile Bioinformatics software, Yale University, version 3.6.1) was used to dichotomise the nuclear POLβ expression into high (*H*-score > 130) and low, and cytoplasmic expression into high (*H*-score > 60) and low according to patient outcome in the pure DCIS cohort [28, 29]. Scoring has been done blindly to clinicopathological data and patient outcome. Approximately 30% of cases were double scored by another pathologist and the discrepant cases were reviewed by both observers and a final score was agreed.

### POLβ siRNA knockdown (KD) in DCIS cells

MCF10DCIS cells were a gift from prof. Vimla band laboratory. MCF10DCIS BC cell line was previously derived from a xenograft originating from premalignant MCF10AT cells injected into SCID mice [30]. Injection of the MCF10DCIS cells into SCID mice results in a predominantly comedo DCIS phenotype [30, 31]. MCF10A, MCF-7 and MDA -MB231 breast cancer cell lines were purchased from ATCC. MCF10DCIS and MCF10A cell lines were cultured in DMEM-F12 supplemented with 10% horse serum, 5 mg/ml insulin, 1 mg/ml cholera toxin, and 100 μg/mL EGFR, 5 mg/mL hydrocortisone and 1% penicillin–streptomycin. MDA-MB231 and MCF-7 cells were cultured in minimum essential amino acids medium supplemented with 10% FBS, 1% penicillin–streptomycin, 1% l-glutamine and 1% nonessential amino acids. POLβ siRNA were obtained from Invitrogen, UK (catalogue no. 4390824, ID: S10776). Briefly, 1 × 10^6^ Cells were seeded in T25 cell culture flasks overnight. siRNA constructs were transfected using Lipofectamine 3000 reagent (Invitrogen, UK) (catalogue no. L3000001) as per the manufacturer’s protocol in Opti-MEM low serum medium (catalogue no. 11058021). Transfection efficiency was confirmed on day 3, day 5 and day 7 using western blotting. Evaluation of N-cadherin, C-MYC, OCT4, NANOG, SOX2, MMP-9 and ALDH1 protein expression was performed by western blotting on extracts of MCF10DCIS POLβ control and MCF10-DCIS POLβ_KD.

### Statistical analysis

Statistical analysis was conducted using SPSS software version 24 (Chicago, IL, USA). Relevant statistical analyses have been carried out based on the distribution of data (parametric or non-parametric). Association between POLβ mRNA expression with clinicopathological parameters and outcome was carried out in the METABRIC database. Chi Square, Mann–Whitney and Kruskal Wallis tests were used to evaluate the correlation of clinicopathological parameters with the expression of POLβ protein level. Wilcoxon signed test was used to compare POLβ protein level in the TDLUs and DCIS. Whilst Mann–Witney was used to compare POLβ protein level in pure DCIS and DCIS component coexistent with IBC. To compare between DCIS component and invasive component in mixed cases, Wilcoxon Signed Ranks Test was used. Log rank and Kaplan Meier tests were used to perform the outcome analysis. *p*-value < 0.05 was considered significant.

Ethical approval was obtained by North West Manchester Ethics Committee under the title “Nottingham Health Science Biobank (15/NW/0685)”.

The reporting recommendation for tumour markers prognostic studies (REMARK) criteria was used in this study.

## Results

### POLβ protein expression

Stained full-face sections (*n* = 10) of DCIS and DCIS coexistent with invasive disease showed a homogenous distribution of POLβ expression which demonstrated the efficiency of using the TMA sections to evaluate POLβ expression. POLβ was expressed in nuclei of the TDLUs and tumour cells with variable intensities. Occasional weak to moderate staining in the cytoplasm of tumour cells was also noticed.

POLβ protein expression showed unimodal, non-parametric distribution amongst the study cohort where the median *H*-score for the nuclear POLβ expression was 130 (range 0–270) and for cytoplasmic expression was 60 (range 0–240). Interestingly, these figures were similar to the cut-off points identified by the X-tile software to stratify the cases according to outcome. Based on these cut-off points, high nuclear POLβ expression was observed in 388/465 cases (83%) whilst low cytoplasmic POLβ expression was observed in 322/465 cases (69%).

### POLβ expression in the mixed DCIS cohort

In the DCIS component in the DCIS-mixed cohort, positive nuclear staining was recognised in 189/198 cases (95%). Furthermore, positive cytoplasmic expression was recognised in 179/198 cases (90%). The median *H*-score for nuclear POLβ expression in the DCIS component was 100 (range 0–220) and the median *H*-score for nuclear POLβ expression in the invasive component was 90 (range 0–180). For the cytoplasmic expression in the DCIS component, the median *H*-score was 60 (range 0–130). Low POLβ nuclear expression was observed in 46 out of 194 cases (76%), whilst low cytoplasmic expression was observed in 139/194 cases (72%) in the DCIS component.

Low nuclear expression was observed in 72 out of 222 cases (32%) of invasive component of the mixed cohort. However, 139/194 cases (72%) representing low cytoplasmic expression were seen in the mixed DCIS coexistent with IBC of the mixed cohort (Fig. 1B–F).

**Fig. 2:**
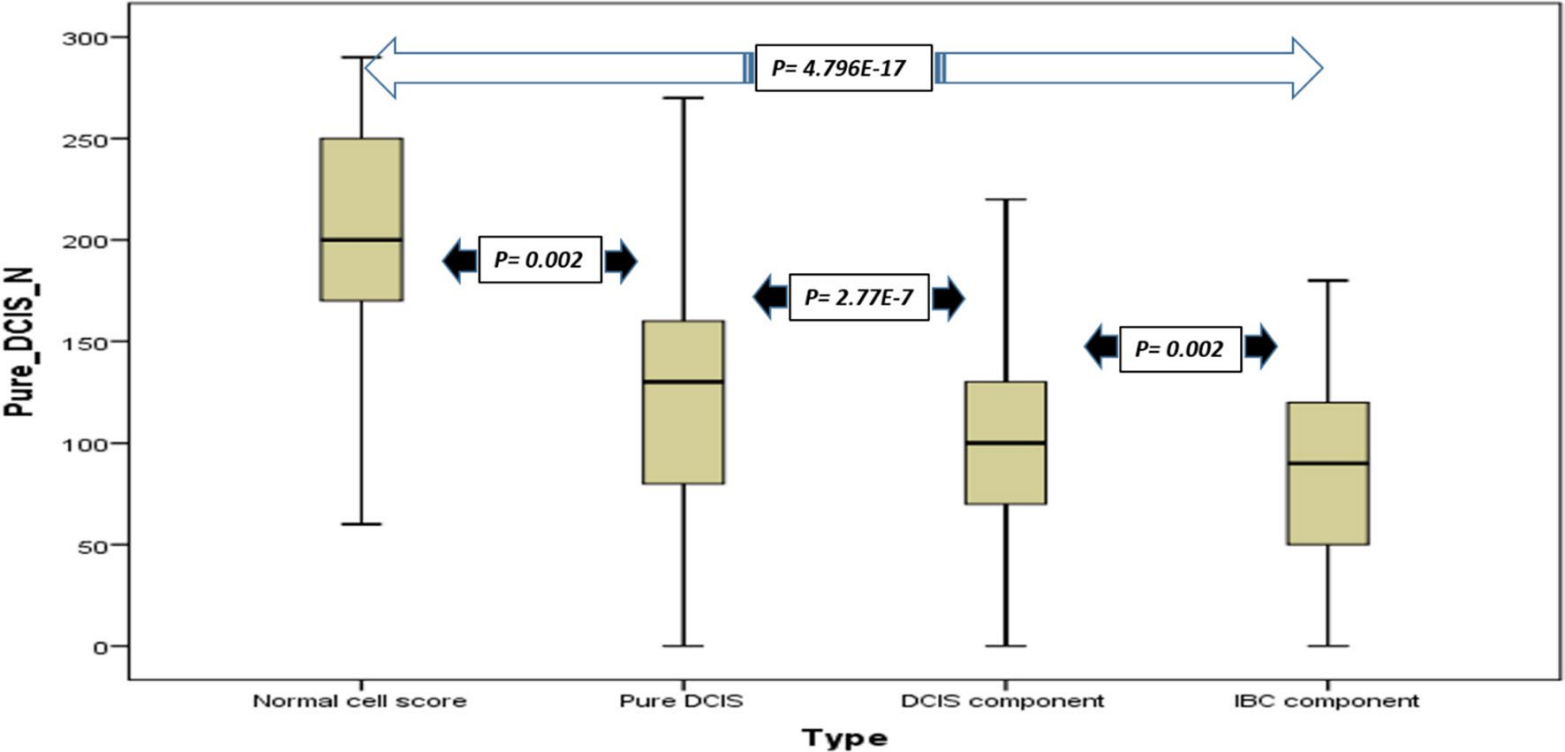
POL-β nuclear protein expression boxplot. POL-β nuclear protein expression boxplot showing the highest level of POL-β nuclear protein expression in the normal TDLUs, decreased to the lowest level in the IBC component of the mixed IBC series

POLβ protein expression levels were higher in the nuclei of TDLUs compared with the pure DCIS (*p* = 0.002). The level of nuclear POLβ expression in the pure DCIS was higher than the DCIS component coexistent with invasive cancer (*p* < 0.001). Moreover, the nuclear POLβ expression in the DCIS component coexistent with invasive in the mixed cohort was higher than the invasive component (*p* = 0.002). The nuclear intensity expression was greater in the TDLUs compare with the invasive component (*p* < 0.001) (Fig. 2).

### The association between POLβ expression and clinicopathological parameters

Low nuclear POLβ protein expression in pure DCIS cohort was associated with aggressive clinicopathological parameters, including high nuclear grade (*p* = 0.001), comedo necrosis (*p* = 0.006), negative hormonal status (*p* < 0.001), positive HER2 status (*p* = 0.005) and with HER2 in molecular subtypes (*p* < 0.001) (Table 1). There was a significant association of low nuclear POLβ expression with large DCIS size (p = 0.018) and high proliferative index Ki-67 (*p* = 0.008) (Supplementary Table S2). Low cytoplasmic POLβ expression was significantly associated with larger DCIS size (*p* = 0.017), diffused type of DCIS (*p* = 0.025) high nuclear grade (*p* = 0.015), PR negativity (*p* = 0.046), HER2 positivity (*p* = 0.007) and higher proliferation index (Ki67*) (*p = 0.021) (Table 2 and Supplementary Table S3).

**Table 1:** Correlation between nuclear POL-β protein expression and clinicopathological parameters in pure DCIS cohort using categorical values

Parameters	Low exp No. (%)	High exp No. (%)	Total No. (%)	(χ^2^) *p*-value
Age (Years)				
≤ 50	20 (17.0)	96 (83.0)	116 (25.0)	(0.052) 0.820
> 50	57 (16.0)	292 (84.0)	349 (75.0)	
Size^a^				
< 16 mm	21 (15.0)	124 (72.0)	145 (31.0)	(1.239) 0.538
16–40 mm	30 (16.0)	154 (84.0	184 (40.0)	
> 40 mm	26 (19.0)	108 (81.0)	134 (29.0)	
DCIS presentation				
Screening	34 (15.0)	192 (85.0)	226 (49.0)	(0.730) 0.393
Symptomatic	43(18.0)	196 (82.0)	239 (51.0)	
Nuclear grade				
Low	4 (7.0)	57 (93.0)	61 (13.0)	(13.391) **0.001**
Moderate	12 (10.0)	109 (90.0)	121 (26.0)	
High	61 (22.0)	222 (78.0)	283 (61.0)	
Comedo necrosis				
No	17 (10.0)	150 (90.0)	167 (39.0)	(7.676) **0.006**
Yes	60 (20.0)	238 (80.0)	283 (61.0)	
Oestrogen status				
Negative	31 (28.0)	81 (72.0)	112 (26.0)	(13.414) ** < 0.001**
Positive	41 (13.0)	281(87.0)	322 (74.0)	
Progesterone status				
Negative	47 (26.0)	135 (74.0)	182 (41.0)	(20.463) ** < 0.001**
Positive	25 (10.0)	234 (90.0)	259 (59.0)	
Her2 status				
Negative	43 (13.0)	281 (87.0)	324 (77.0)	(8.068) **0.005**
Positive	25 (25.0)	74 (75.0)	99 (23.0)	
Proliferation index (Ki 67)				
Low (< 14%)	47 (15.0)	266 (85.0)	313 (77.0)	(2.191) 0.139
High (≥ 14%)	20 (22.0)	73 (78.0)	93 (23.0)	
Molecular classes				
Luminal A	21 (11.0)	170 (89.0)	191 (51.0)	**(18.926)** ** < 0.001**
Luminal B	11 (14.0)	69 (86.0)	80 (21.0)	
Her2	16 (37.0)	27 (63.0)	43 (11.0)	
Triple negative	12 (20.0)	84 (80.0)	60 (16.0)	

Significant *p*-values are in bold

*No* number, *χ*^*2*^ Chi square, *POL-β* DNA polymerase beta, *DCIS* ductal carcinoma in situ, *HER2* human epidermal growth factor receptor 2

^a^Size: based on Van Nuys Prognostic Index (VNPI)

**Table 2:** Correlation between cytoplasmic POL-β expression in DCIS with clinicopathological parameters in pure DCIS cohort using categorical values

Parameters	Low exp No. (%)	High exp No. (%)	Total No. (%)	(χ^2^) p-value
Age (years)				
≤ 50	86 (74.0)	30 (56.0)	116 (25.0)	(1.736) 0.188
> 50	236 (6.0)	113 (32.0)	349 (75.0)	
Size^a^				
< 16 mm	88 (61.0)	57 (39.0)	145 (31.0)	(8.169) **0.017**
16–40 mm	133 (72.0)	51 (28.0)	184 (40.0)	
> 40 mm	101 (75.0)	33 (25.0)	134 (29.0)	
DCIS presentation				
Screening	159 (70.0)	67 (30.0)	226 (49.0)	(0.253) 0.615
Symptomatic	163 (68.0)	76 (32.0)	239 (51.0)	
Nuclear grade				
Low	37 (61.0)	24 (39.0)	61 (13.0)	(8.379) **0.015**
Moderate	75 (62.0)	46 (38.0)	121 (26.0)	
High	210 (74.0)	73 (26.0)	283 (61.0)	
Comedo necrosis				
No	109 (65.0)	58 (35.0)	167 (36.0)	(1.936) 0.164
Yes	21 (71.0)	85 (29.0)	298 (64.0)	
Oestrogen status				
Negative	84 (75.0)	28 (25.0)	112 (26.0)	(3.013) 0.083
Positive	213 (66.0)	109 (34.0)	322 (74.0)	
Progesterone status				
Negative	135 (74.0)	47 (26.0)	182 (41.0)	(3.976) **0.046**
Positive	169 (65.0)	90 (35.0)	259 (59.0)	
Her2 status				
Negative	212 (65.0)	112 (35.0)	324 (77.0)	(7.290) **0.007**
Positive	79 (80.0)	20 (20.0)	99 (23.0)	
Proliferation index (Ki 67)				
Low (< 14%)	210 (67.0)	103 (33.0)	313 (77.0)	(5.310) **0.021**
High (≥ 14%)	74 (80.0)	19 (20.0)	93 (23.0)	
Molecular classes				
Luminal A	127 (66.0)	64 (34.0)	191 (51.0)	(6.852) 0.077
Luminal B	55 (69.0)	25 (31.0)	80 (21.0)	
Her2	37 (86.0)	6 (14.0)	43 (12.0)	
Triple negative	39 (65.0)	21 (35.0)	60 (16.0)	

Significant *p*-values are in bold

*No* number, *χ*^*2*^ Chi square, *POL-β* DNA polymerase beta, DCIS ductal carcinoma in situ, *HER2* Human epidermal growth factor receptor 2, *LCIS* lobular carcinoma in situ, *BCS* Breast conserving

^a^Size: based on Van Nuys Prognostic Index (VNPI)

Low POLβ nuclear expression in DCIS-mixed cohort was significantly associated with larger tumour size (*p* = 0.022), high nuclear grade (*p* = 0.025), comedo necrosis (*p* = 0.002), negative oestrogen receptor (*p* = 0.001) and with patients treated with mastectomy (*p* = 0.023). Results were confirmed by continuous data analysis (Table 3). Additionally, low cytoplasmic POLβ expression was associated with high nuclear grade (*p* = 0.034), presence of comedo necrosis (0.018) and negative oestrogen receptor (*p* = 0.018). Similar results were obtained by analysis of continuous data (Table 4).

**Table 3:** Correlation between POL-β nuclear protein expression in DCIS component in mixed cohort

Parameters	Categorical values			Continuous values			
	Low exp No. (%)	High exp No. (%)	Total No. (%)	(χ^2^) *p*-value	No. of cases	Mean rank	(χ^2^) *p*-value
Age (years)							
≤ 50	23 (25.0)	71 (75.0)	94 (48.0)	0.058	94	94.22	0.429
> 50	23 (23.0)	77 (77.0)	100 (52.0)	0.810	100	100.58	
Size^a^							
< 16 mm	19 (22.0)	69 (78.0)	88 (45.0)	**7.678**	88	101.53	0.197
16–40 mm	21 (22.0)	75 (78.0)	96 (50.0)	**0.022**	96	96.88	
> 40 mm	6 (60.0)	4 (40.0)	10 (5.0)		10	67.95	
Nuclear grade							
Low	1 (10.0)	9 (90.0)	10 (5.0)	**7.355**	10	137.75	** < 0.001**
Moderate	5 (11.0)	41 (89.0)	46 (24.0)	**0.025**	46	119.10	
High	40 (29.0)	98 (71.0)	138(71.0)		138	87.38	
Comedo necrosis							
No	3 (7.0)	42 (93.0)	45 (23.0)	**9.410**	45	132.66	** < 0.001**
Yes	43 (29.0)	106(71.0)	149(77.0)	**0.002**	149	86.88	
Oestrogen status							
Negative	10 (53.0)	9 (47.0)	19 (10.0)	**10.130**	19	59.42	**0.002**
Positive	35 (20.0)	139 (80.0)	174 (90.0)	**0.001**	174	101.10	
Final operation							
Mastectomy	29 (31.0)	65 (69.0)	94 (48.0)	**5.139**	94	88.63	**0.032**
BCS	17 (17.0)	83 (83.0)	100(52.0)	**0.023**	100	105.84	

Significant *p*-values are in bold

*No* number, *χ*^*2*^ Chi square, *POL-β* DNA polymerase beta, *DCIS* ductal carcinoma in situ, *BCS* breast conserving, *BCS* breast conserving surgery

^a^Size: based on Van Nuys Prognostic Index (VNPI)

**Table 4:** Correlation between POLβ cytoplasmic protein expression in DCIS component in mixed cohort

Parameters	Categorical values				Continuous values		
	Low exp No. (%)	High exp No. (%)	Total No. (%)	(χ^2^) *p* value	No. of cases	Mean rank	(χ^2^) *p* value
Age (years)							
≤ 50	69 (73.0)	25 (27.0)	94 (48.0)	0.058	94	93.61	0.346
> 50	70 (70.0)	30 (30.0)	100(52.0)	0.810	100	101.16	
Size^a^							
< 16 mm	59 (67.0)	29 (33.0)	88 (45.0)	2.828	88	100.02	0.364
16–40 mm	71 (74.0)	25 (26.0)	96 (50.0)	0.243	96	97.68	
> 40 mm	9 (90.0)	1 (10.0)	10 (5.0)		10	73.55	
Nuclear grade							
Low	6 (60.0)	4 (40.0)	10 (5.0)	**6.278**	10	113.65	**0.013**
Moderate	27 (27.0)	19 (41.0)	46 (24.0)	**0.034**	46	116.45	
High	106(77.0)	32 (23.0)	138(71.0)		138	90.01	
Comedo necrosis							
No	26 (58.0)	19 (42.0)	45 (25.0)	**5.550**	45	117.66	**0.006**
Yes	113(76.0)	36 (24.0)	149(75.0)	**0.018**	149	91.41	
Oestrogen status							
Negative	18 (95.0)	1 (5.0)	19 (10.0)	**5.583**	19	81.00	0.186
Positive	120(69.0)	54(31.0)	174(90.0)	**0.018**	174	98.75	
Final operation							
Mastectomy	73 (78.0)	21 (22.0)	94 (48.0)	3.243	94	88.63	**0.032**
BCS	66(66.0)	34 (34.0)	100(52.0)	0.072	100	105.84	

Significant *p*-values are in bold. Mean rank operated by MannWhitey test and Kurskal test

*POL-β* DNA polymerase beta, *DCIS* ductal carcinoma in situ, *Her2* human epidermal growth factor receptor 2, *BC*S breast conserving

^a^Size: based on Van Nuys Prognostic Index (VNPI)

POLβ nuclear/cytoplasmic (N/C) protein co-expression has been investigated in the DCIS cohort. 29% demonstrated high nuclear/high cytoplasmic expression (H.N/H.C), 54% showed H.N/L.C, 15% with L.N/L.C and 2% demonstrated L.N/H.C.

The L.N/L.C cluster was significantly associated with aggressive behaviour including high nuclear grade (*p* = 0.003), presence of comedo necrosis (*p* = 0.026), and larger size of DCIS (*p* = 0.010). However, the low protein expression of POLβ (L.N/L.C cluster) was observed more in the ER positive (*p* = 0.001) and HER2 negative (p = 0.004) tumours and in the luminal A molecular subgroup compared to other molecular subgroups (*p* < 0.001) (Table 5).

**Table 5:** The correlation between Nuclear/Cytoplasmic (clustering) POLB expression in pure DCIS cohort with clinicopathological parameters

Parameters	H.N/H.C No. (%)	H.N/L.C No. (%)	L.N/L.C No. (%)	L.N/ H.C No. (%)	Total No. (%)	(χ2) *p* value
Age (years)						
≤ 50	28 (21.0)	68 (27.0)	18 (26.0)	2 (25.0)	116 (25.0)	(1.827) 0.585
> 50	107 (79.0)	185 (73.0)	51 (74.0)	6 (75.0)	349 (75.0)	
DCIS size						
≤ 20 mm	74 (56.0)	98 (39.0)	31 (45.0)	5 (62.0)	208 (45.0)	**(11.087)** **0.010**
> 20 mm	59 (44.0)	155 (61.0)	38 (55.0)	3 (38.0)	255 (55.0)	
DCIS presentation						
Screening	63 (47.0)	129 (51.0)	30 (44.0)	4 (50.0)	226 (49.0)	(1.510) 0.673
Symptomatic	72 (53.0)	124 (49.0)	39 (56.0)	4 (50.0)	239 (51.0)	
Nuclear grade						
Low	24 (18.0)	33 (13.0)	4 (6.0)	0 (0.0)	61 (13.0)	(18.978) **0.003**
Moderate	44 (33.0)	65 (26.0)	10 (14.0)	2 (52.0)	121 (26.0)	
High	67 (50.0)	155 (61.0)	55 (80.0)	6 (75.0)	283 (61.0)	
Comedo necrosis						
No	57 (42.0)	93 (37.0)	16 (23.0)	1 (13.0)	167 (36.0)	(9.173) **0.026**
Yes	78 (58.0)	160 (63.0)	53 (77.0)	7 (88.0)	298 (64.0)	
Oestrogen receptor						
Negative	23 (18.0)	58 (25.0)	26 (41.0)	5 (62.0)	112 (26.0)	(17.354) **0.001**
Positive	106 (82.0)	175 (75.0)	38 (59.0)	3 (38.0)	322 (74.0)	
Her2 status						
Negative	107(86.0)	174 (76.0)	38 (62.0)	5 (71.0)	324 (77.0)	(12.831) **0.004**
Positive	18 (14.0)	56 ( 24.0)	23 (38.0)	2 (29.0)	99 (23.0)	
Proliferation index (Ki 67						
Low (≤ 14%)	98 (84.0)	168 (75.0)	42 (69.0)	5 (83.0)	313 (77.0)	(6.455) 0.078
High (> 14%)	18 (16.0)	55 (25.0)	19 (31.0)	1 (17.0)	93 (23.0)	
Molecular classes						
Luminal A	64 (58.0)	106 (52.0)	21 (39.0)	0 (0.0)	191 (51.0)	(33.130) ** < 0.001**
Luminal B	24 (22.0)	45 (22.0**)**	10 (18.0)	1 (17.0)	80 (21.0)	
Her2	5 (5.0)	22 (11.0)	15 (27.0)	1 (17.0)	43 (11.0)	
Triple negative	17 (16.0)	31 (15.0)	8 (15.0)	4 (66.0)	60 (16.0)	

Significant *p*-values are in bold

*POLβ* DNA polymerase beta, *DCIS* ductai carcinoma in situ, *HER2 Enriched* human epidermal growth factor receptor 2, *H.N/H.C* high nuclear/low cytoplasmic expression, *H.N/L.C* high nuclear/low cytoplasmic expression, *L.N/L.C* low nuclear/low cytoplasmic expression, *L.N/H.C* low nuclear/high cytoplasmic expression

### METABRIC cohort

**Fig. 3:**
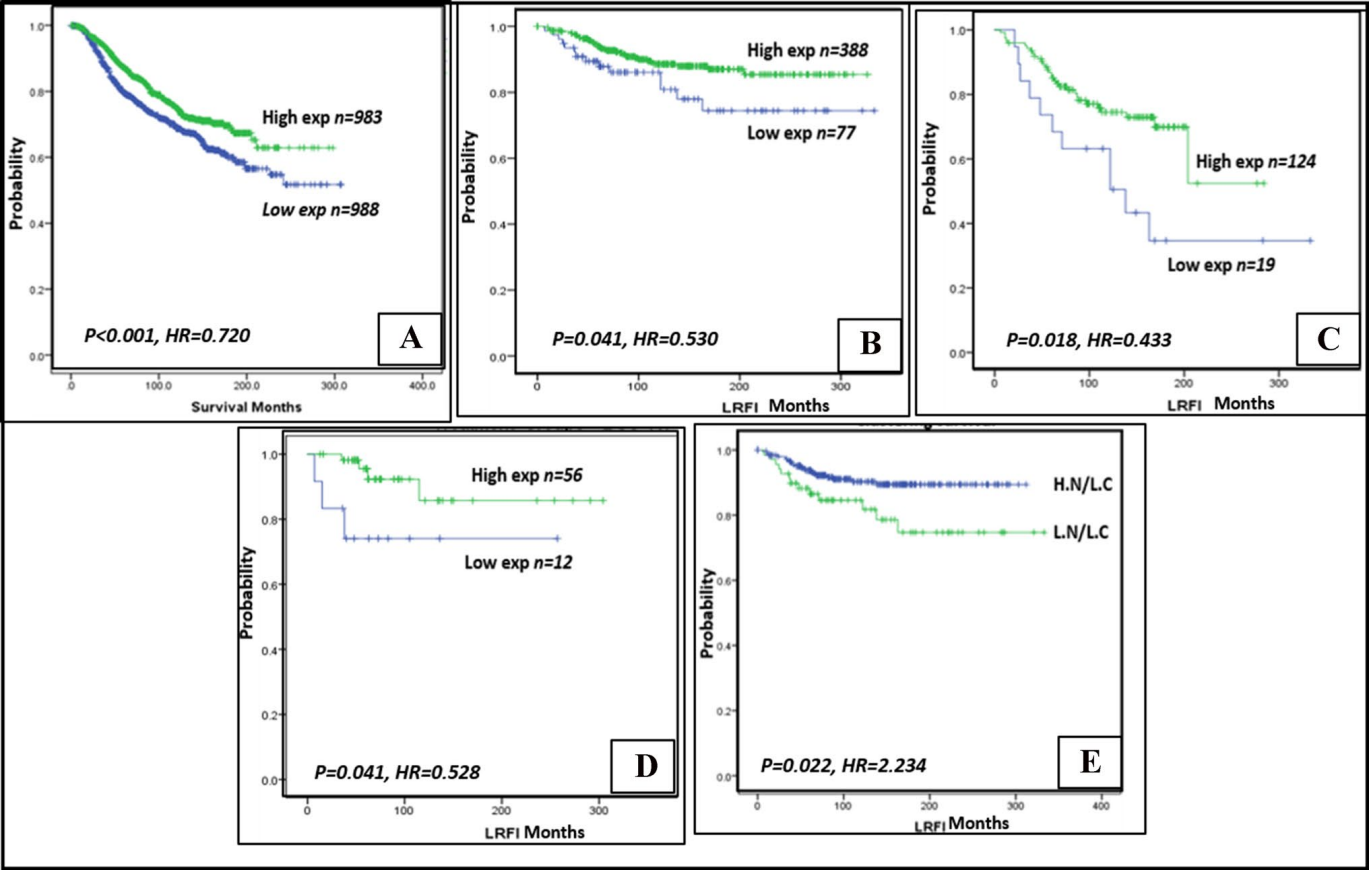
Association between POL-β expression and outcome. Kaplan–Meier curves showing low expression of POL-β nuclear protein expression in tumour breast epithelial cells associated with **A** shorter LRFI in all recurrences of pure DCIS cohort. **B** In patients treated with BCS only. **C** In patients received adjuvant radiotherapy after BCS. **D** Shorter breast cancer specific survival of IBC in the METABRIC cohort. **E**, **F** Nuclear/cytoplasmic analysis revealed L.N/L.C cluster associated with shorter LRFI

Low *POLβ* mRNA expression was associated with young patient age (*p* = 0.001), premenopausal status (*p* = 0.015), high tumour grade (*p* < 0.001), negative hormonal status (ER&PR) (*p* < 0.001), positive HER2 status (*p* < 0.001) and basal-like breast cancer molecular subtype (*p* < 0.001) (Supplementary Table S4). Moreover, low *POLβ* mRNA expression was associated with shorter BCSS (*p* < 0.001, HR = 0.720, 95% CI 0.604–0.859) (Fig. 3A).

### Association of POLβ and patient outcome

Low nuclear expression of POLβ in the DCIS was significantly associated with poor outcome in the form of shorter LRFI for all recurrences (in situ recurrence and invasive recurrence) (*p* = 0.041. HR = 0.530, 95% CI 0.287–0.976) (Fig. 3B). A significant association was observed in patients who underwent BCS without adjuvant radiotherapy (RT) (*p* = 0.018. HR = 0.433, 95% CI 0.217–0.865), as well as in patients treated with BCS and received adjuvant radiotherapy (BCS + RT) (*p* = 0.041. HR = 0.528, 95% CI 0.287–0.973) (Fig. 3C and D respectively).

Although POLβ cytoplasmic expression in pure DCIS did not show significant associations with patient outcome, combined analysis of N/C expression revealed that L.N/L.C cluster was associated with shorter LRFI (*p* = 0.022, HR = 2.234, 95% CI 1.125–4.436) (Fig. 3E).

Low expression of POLβ nuclear protein in DCIS was an independent predictor of a poor outcome in DCIS when plotted against patient age, DCIS size, nuclear grade, radio-therapeutic treatment, HER2 status and proliferation index Ki67 (*p* = 0.031. HR = 0.490, 95% CI 0.256–0.936) (Table 6). Cytoplasmic POLβ expression but did not reveal any significant association as a predictor of patient outcome.

**Table 6:** Cox regression analysis of POL-β nuclear protein expression in terms of predicting the outcome of local recurrence-free interval (LFRI) in DCIS patients of pure DCIS series treated by breast conserving surgery (BCS)

Parameters	*p*-value	HR	95% CI	
			Lower	Upper
POL-β Expression	**0.031**	0.490	0.256	0.936
Age	0.130	1.599	0.871	2.934
Nuclear Grade	0.678	1.096	0.710	1.694
Radiotherapy	0.893	0.942	0.392	2.262
Her2 Status	0.730	0.881	0.429	1.810
Proliferation index (Ki 67)	0.956	0.979	0.453	2.114

All recurrence /Multivariate survival analysis. Significant *p*-values are in bold

*HR* Hazard ratio, *CI* confidence interval, *POL-β* DNA polymerase beta, *DCIS* ductal carcinoma in situ, Her2 human epidermal growth factor receptor 2

### Functional studies

**Fig. 4:**
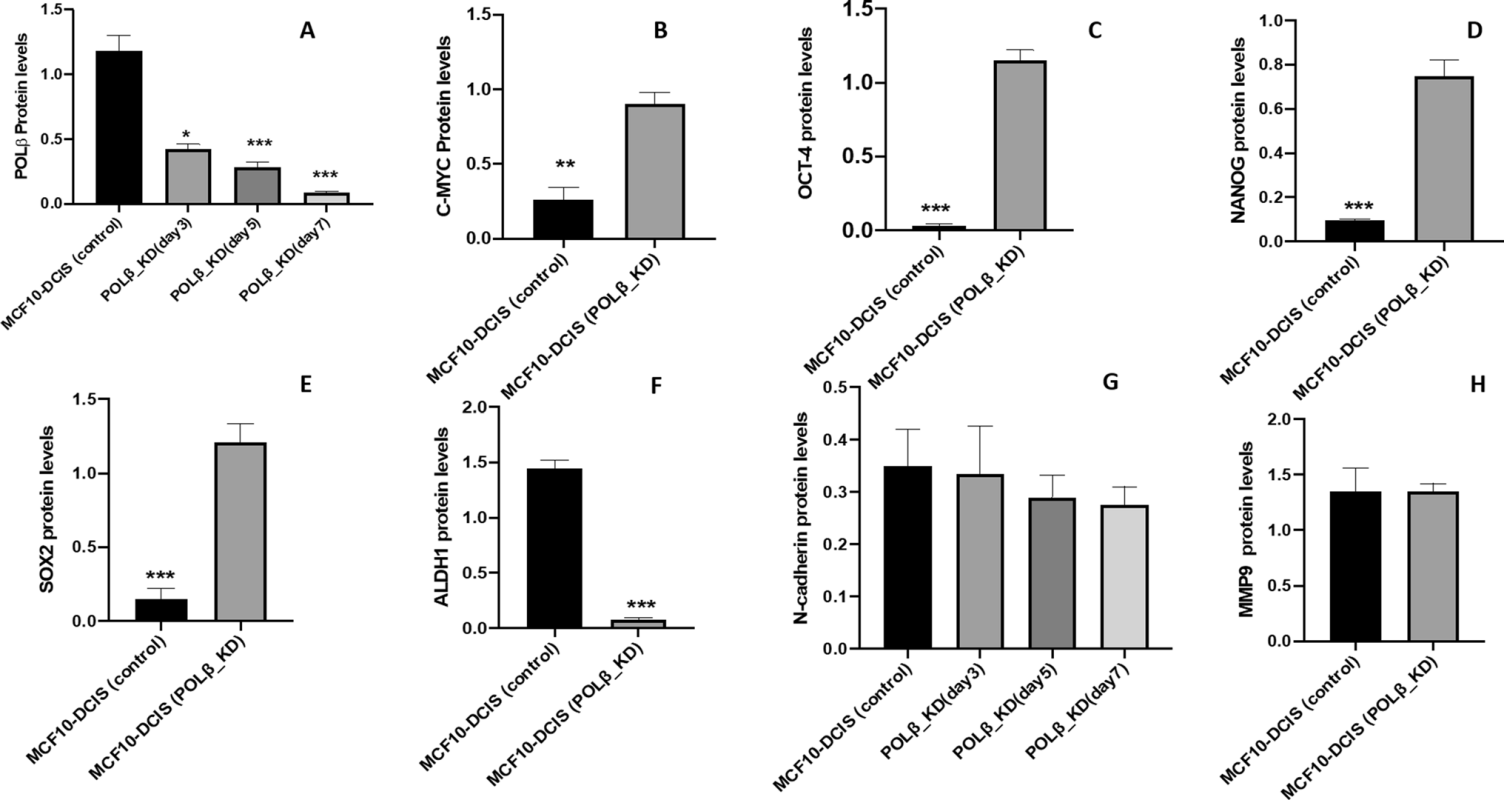
POL-β Knockdown and Mechanistic in vitro functional results. Demonstrate POL-β gene KD in day 3, 5 and 7 compared with the control (**A**). Expression of C-MYC, OCT4, NANOG, SOX2, ALDH1, N-cadherin and MMP-9 protein levels were demonstrating in MCF10-DCIS_POLβ _KD compared with the control MCF10-DCIS (**B**–**H** respectively). *p*-values representation: (*) equal to *p*-values *p* ≤ 0.05, (**) equal to *p* ≤ 0.01 and (***) equal to *p* ≤ 0.001

POLβ depletion and stemness phenotype in MCF10-DCIS cell line: The clinical data shown here suggest that downregulation of POLβ is associated with aggressive breast cancer pathogenesis. Therefore, we hypothesised that POLβ depletion could be associated with increased stemness in DCIS leading to aggressive phenotype. We showed that MCF10-DCIS cells are non-invasive cell line, similar to MCF10A non-cancerous epithelial cells [32]. We performed POLβ knockdown by siRNA in MCF10-DCIS cells. We observed a robust knockdown of POLβ in our cells in day 3, 5 and 7, the most depleted level of POLβ protein level was observed in day 7 (Fig. 4A). We evaluated the expression of well identified stem cells markers in MCF10DCIS_POLβ_KD cells compared to controls. Interestingly, MCF10-DCIS_POLβ_KD cells have a noticeable increase in C-MYC, OCT4, NANOG and SOX2 protein expression (Fig. 4B–E respectively) suggesting that MCF10-DCIS_POLβ_KD acquired stemness phenotype associated cancerous self-renewal and increased cell division. However, MCF10-DCIS_POLβ_KD cells has a noticeable decrease in ALDH1 and no changes in N-cadherin and MMP-9 protein levels were observed in MCF10-DCIS_POLβ_KD cells (Fig. 4F–H respectively).

## Discussion

Although breast cancer is a heterogeneous disease, there is a great similarity between DCIS and invasive carcinoma at histological and molecular levels. DNA damage repair is a complex mechanism that depends on the interaction between the various pathways to repair impaired DNA. The mechanisms of DNA repair act as a barrier to maintain genetic stability as well as preventing cancer development. Following DNA damage, one or more repair pathways are activated, such as BER. POLβ is one of the most important DNA polymerases in BER as it contributes to genome stability maintenance [16, 33]. The role of the POLβ gene has been studied in many different tumours [16, 17, 27, 34–37]. In this study, POLβ low expression in the METABRIC cohort showed a significant association with an aggressive phenotype. This indicates the tumour suppressor role of POLβ in BC.

This study aimed to evaluate the expression of POLβ in a large DCIS cohort, as well as a DCIS-mixed cohort. The data provide evidence that POLβ might have a crucial role in DCIS genomic stability. Starcevic et al. [38] reported that one-third of total tumours examined expressed POLβ variant proteins and could induce genomic instability. Our data showed an association between low POLβ expression and aggressive DCIS phenotypes such as a high nuclear grade, which is consistent with Chantre-Justino et al. [33]. BER transcript profiling based on grade demonstrated differences in the molecular signature between the high and low-grade tumours, which is referred to as differential transcriptional regulation suggesting that BER dysregulation could promote carcinogenesis. POLβ gene has a misalignment-mediated mutator activity associated with aggressive mutator phenotype [38, 39]. It has been reported that cells with reduced expression of POLβ protein accumulate DNA damage and aggressive cancerous phenotype may be driven by mutation and genomic instability that result from impaired BER [40]. Moreover, increased POLβ expression proved to be resistant to DNA damage. These results suggest that POLβ can act as a caretaker gene where its absence associates with aggressive behaviour [41]. Our results showed that negative ER expression is associated with aggressive behaviour. Mobbley and Brueggemeier [42] reported that oestrogen evolves to carcinogenesis in the breast by creating a link that connects oestrogen-induced BC and an oxidative stress pathway. Moreover, there is a robust relationship between ER responsiveness and oxidative DNA damage that is significantly elevated by approximately ten-fold in the invasive BC compared to normal breast tissue and about three-fold higher in positive ER than negative ER BC [43]. Bhat et al. [44] mentioned that oestrogen could promote carcinogenicity by inducing oxidative stress. Interestingly, our results showed that low nuclear POLβ level was associated with ER negative cases which probably suggests that non oestrogen mediated oxidative stress pathways trigger DNA damage and particularly involve in DCIS.

Following our result, it has been found that amplified Her2 was associated significantly with aggressive behaviour and poor prognosis in breast cancer [25, 45]. Notably, expression of the HER2 level was different within various stages of BC progression. In general, Her2 in TDLUs was rarely detected in atypical ductal hyperplasia (ADH) but was amplified or overexpressed in high nuclear grade DCIS, especially in types of comedo necrosis and in a high nuclear grade of IBC. A loss of, or undetectable, Her2 protein levels in benign lesions suggests that its amplification or overexpression occurs in the transition from hyperplasia to DCIS, suggesting that overexpression is considered significant in early malignant progression [46]. It is therefore noticeable that the trend of decreasing POLβ occurs simultaneously with increasing HER2 expression when tumour aggressiveness increases. However, it is hard to conclude whether there is direct or indirect crosstalk between these two proteins as further molecular investigations required.

In this study, we explored the level of POLβ nuclear protein in a comparative cohort (mixed DCIS/IBC cohort). Our observations showed that a decreasing trend of POLβ levels was demonstrated starting from TDLUs, pure DCIS series, DCIS component coexisting with invasive disease and invasive component which showed the lowest level of POLβ protein in the study cohort. This observation supports the notion of our hypothesis which states that the lack, or loss, of the POLβ protein associates with aggressive DCIS phenotype.

Low POLβ nuclear protein level showed significant association with shorter LRFI. A low level of nuclear POLβ was associated with recurrence in patients treated with adjuvant radiotherapy and in patients who did not receive radiotherapeutics treatment after BCS. This is a clear signal suggesting that radio-therapeutic treatment does not provide any advantages in DCIS patients with a low level of POLβ nuclear protein. More functional studies are required to understand the roles of POLβ in DCIS, especially with treatment. In clustering N/C survival, our data showed that the L.N/L.C cluster was the worst group in patient outcomes based on LRFI survival, this observation agrees with the aggressive attribute of low POLβ in DCIS.

Low POLβ nuclear protein was an independent predictor for all recurrences. Additionally, our preliminary study of POLβ depletion in MCF10-DCIS cells suggests that loss of POLβ could be associated with increased stemness phenotype in DCIS and hence progression to invasive breast cancer. POLβ knockdown cells had a significant increase in NANOG, SOX2, C-MYC and OCT4 which are well-known markers of cancer stem cells. However, overexpression of those markers was not associated with epithelial mesenchymal transition (EMT) as evidence by the no change in N-cadherin and MMP-9 which might have been more significant in a POLβ stable knock out cell line model. We anticipate that those changes happen over time DCIS. Therefore, more work is needed to identify the link between POLβ and stemness phenotype.

The study has some limitations it was carried out using TMA tissue preparations that may underestimate DCIS intratumour heterogeneity. However, full-face tissue sections revealed complete comparability of the heterogeneity of IHC expression. Moreover, the study included a small number of recurrence cases, limited data of patients with recurrence cases who received systemic therapy.

In conclusion, this study has shown the potential role of POLβ as a caretaker and tumour suppressor gene. The data provide evidence that loss or reduced expression of POLβ promotes tumour progression and is most probably associated with the aggressive behaviour of DCIS, which could progress to an invasive stage. Moreover, low POLβ protein level was also associated with a poor outcome within the DCIS. However, further molecular studies are required to further understand the underlying mechanisms.

## Supplementary Information

Below is the link to the electronic supplementary material.

Supplementary file1 (DOCX 42 kb)

## Data Availability

The authors confirm the data that has been used in this work are available on reasonable request.
