# Supplementary material for: The frequency and clinical significance of DNA polymerase beta (POLβ) expression in breast ductal carcinoma in situ (DCIS)
Source: Breast Cancer Res Treat. 2021 Aug 18;190(1):39–51. doi: 10.1007/s10549-021-06357-7 (PMC8557137; doi:10.1007/s10549-021-06357-7)
Supplement: Supplementary file 1 — Supplementary file1 (DOCX 42 kb) [file 10549_2021_6357_MOESM1_ESM.docx]

**Supplementary**

**Supplementary table S1:-** Patient demographic in pure DCIS cohort

| Clinicopathological parameter | Groups | Number of patients & (%) |
| --- | --- | --- |
| Age_50 | ≤50 | 232 (22%) |
|  | >50 | 827 (78%) |
| Method of Presentation | Screening | 606 (57%) |
|  | Symptomatic | 453 (43%) |
| DCIS Size (mm) | ≤20mm | 542 (52%) |
|  | >20mm | 510 (48%) |
| Nuclear Grade | Low | 143 (14%) |
|  | Intermediate | 268 (25%) |
|  | High | 644 (61%) |
| Associated with Comedo Necrosis | Yes | 695 (66%) |
|  | No | 364 (34%) |
| Radiotherapy (RT) | Yes | 181 (17%) |
|  | No | 878 (83%) |
| Treatment group | Mastectomy | 542 (51%) |
|  | Breast conserving surgery (BCS) | 340 (32%) |
|  | Breast conserving surgery +Radiotherapy (RT) | 176 (17%) |
| Recurrence | Yes | 95 (9%) |
|  | No | 964 (91%) |
| Oestrogen status | Negative | 156 (22%) |
|  | Positive | 545 (78%) |
| Progesterone status | Negative | 260 (43%) |
|  | Positive | 340 (57%) |
| Her2 status | Negative | 517 (80%) |
|  | Positive | 131 (20%) |
| Proliferation index (Ki 67) | Low proliferation | 389 (77%) |
|  | High proliferation | 118 (23%) |
| Molecular classes | Luminal A | 248 (52%) |
|  | Luminal B | 88 (19%) |
|  | Her2 | 64 (14%) |
|  | Triple negative | 73 (15%) |

DCIS: Ductal carcinoma in situ, HER2; Human epidermal growth factor receptor

**Supplementary table S2:** Correlation between nuclear POL-β expression in DCIS with clinicopathological parameters in pure DCIS cohort using continuous values.

| *Parameters* | *No. of cases* | *Mean Rank* | *p-value* |
| --- | --- | --- | --- |
| *Age*  *≤50*  *>50* | *116*  *349* | *227.53*  *234.82* | *0.612* |
| *Size**  *<16mm*  *16-40mm*  *>40mm* | *145*  *184*  *134* | *254.52*  *230.88*  *209.17* | ***0.018*** |
| *DCIS Presentation*  *Screening*  *Symptomatic* | *226*  *239* | *228.79*  *237.98* | *0.511* |
| *Nuclear Grade*  *Low*  *Moderate*  *High* | *61*  *121*  *283* | *289.02*  *282.54*  *199.74* | ***<0.001*** |
| *Comedo Necrosis*  *No*  *Yes* | *167*  *298* | *270.54*  *211.96* | ***<0.001*** |
| *LCIS*  *No*  *Yes* | *425*  *40* | *228.36*  *282.34* | ***0.015*** |
| *Oestrogen Status*  *Negative*  *Positive* | *112*  *322* | *158.89*  *237.89* | ***<0.001*** |
| *Progesterone Status*  *Negative*  *Positive* | *182*  *259* | *175.99*  *252.63* | ***<0.001*** |
| *Her2 Status*  *Negative*  *Positive* | *324*  *99* | *223.98*  *172.80* | ***<0.001*** |
| *Proliferation index (Ki 67)*  *Low (<14%)*  *High (≥14%)* | *257*  *93* | *184.18*  *151.51* | ***0.008*** |
| *Molecular classes*  *Luminal A*  *Luminal B*  *Her2*  *Triple Negative* | *191*  *80*  *43*  *60* | *214.09*  *190.32*  *120.55*  *147.09* | ***<0.001*** |

Significant p values are in bold, No: Number, X2: Chi square. POL-β; DNA polymerase beta, DCIS; Ductal Carcinoma in Situ, HER2; Human epidermal growth factor receptor 2. LCIS: Lobular Carcinoma in Situ, BCS: Breast conserving. * Size: based on Van Nuys Prognostic Index (VNPI).

**Supplementary table S3;** Correlation between cytoplasmic POL-β expression in DCIS with clinicopathological parameters in pure DCIS cohort using continuous values.

| *Parameters* | *No. of cases* | *Mean Rank* | *p-value* |
| --- | --- | --- | --- |
| *Age*  *≤50*  *>50* | *116*  *349* | *220.71*  *237.08* | *0.252* |
| *Size**  *<16mm*  *16-40mm*  *>40mm* | *145*  *184*  *134* | *255.89*  *222.13*  *219.70* | ***0.033*** |
| *DCIS Presentation*  *Screening*  *Symptomatic* | *226*  *239* | *233.48*  *232.55* | *0.940* |
| *Extent*  *Localised*  *Diffuse* | *319*  *66* | *199.96*  *159.34* | ***0.007*** |
| *Nuclear Grade*  *Low*  *Moderate*  *High* | *61*  *121*  *283* | *266.24*  *257.47*  *215.37* | ***0.002*** |
| *Comedo Necrosis*  *No*  *Yes* | *167*  *298* | *252.35*  *222.16* | ***0.019*** |
| *Oestrogen Status*  *Negative*  *Positive* | *112*  *322* | *196.87*  *224.68* | ***0.042*** |
| *Progesterone Status*  *Negative*  *Positive* | *182*  *259* | *201.09*  *234.99* | ***0.006*** |
| *Her2 Status*  *Negative*  *Positive* | *324*  *99* | *223.16*  *175.48* | ***0.001*** |
| *Proliferation index (Ki 67)*  *Low (<14%)*  *High (≥14%)* | *257*  *93* | *181.93*  *157.73* | ***0.046*** |
| *Molecular classes*  *Luminal A*  *Luminal B*  *Her2*  *Triple Negative* | *191*  *80*  *43*  *60* | *196.30*  *183.66*  *144.62*  *195.34* | ***0.036*** |

Significant *p* values are in **bold,** No: Number, X^2^: Chi square

POL-β; DNA polymerase beta, DCIS; Ductal Carcinoma *in Situ*, HER2; Human epidermal growth factor receptor 2. LCIS: Lobular Carcinoma *in Situ*, BCS: Breast conserving. * Size: based on Van Nuys Prognostic Index (VNPI).

**Supplementary table S4:-** Correlation between POLβ mRNA in METABRIC cohort with clinicopathological parameters in IBC.

| *Parameters* | *Low Exp.*  *No. (%)* | *High Exp.*  *No. (%)* | *Total* | *(χ^2^ )*  *P value* |
| --- | --- | --- | --- | --- |
| *Frequencies*  *Invasive Breast*  *Cancer* | *990 (50.2)* | *984 (50.8)* | *1980* |  |
| *Age*  *≤50*  *>50* | 242 (57.0)  724 (48.0) | 180 (43.0)  783 (52.0) | 422  1507 | **11.414**  **0.001** |
| *Size*  *≤20mm*  *>20mm* | 415 (49.0)  555 (51.0) | 434 (51.0)  543 (49.0) | 849  1098 | 0.531  0.466 |
| *Menopausal*  *Premenopausal*  *Postmenopausal* | 239 (55.0)  743 (49.0) | 194 (49.0)  787 (51.0) | 433  1530 | **5.942**  **0.015** |
| *Nuclear Grade*  *Low*  *Moderate*  *High* | 70 (41.0)  340 (44.0)  531 (56.0) | 100 (59.0)  426 (56.0)  419 (44.0) | 170  766  950 | **28.145**  **<0.001** |
| *Estrogen Status*  *Negative*  *Positive* | 337 (71.0)  653 (44.0) | 136 (29.0)  484 (56.0) | 473  1501 | 110.7  **<0.001** |
| *Progesterone Status*  *Negative*  *Positive* | 561 (60.0)  429 (41.0) | 376 (40.0)  608 (59.0) | 937  1037 | 67.406  **<0.001** |
| *Her2 Status*  *Negative*  *Positive* | 839 (49.0)  151 (61.0) | 889 (51.0)  95 (39.0) | 1728  246 | **14.177**  **<0.001** |
| *Molecular classes*  *Normal like*  *Luminal A*  *Luminal B*  *Basal like*  *Her2* | 138 (70.0)  283 (40.0)  196 (40.0)  218 (66.0)  152 (63.0) | 60 (30.0)  432 (60.0)  290 (60.0)  111 (44.0)  88 (37.0) | 198  715  486  329  240 | 131.808  **<0.001** |

Significant *p* values are in **bold.** No: Number, X^2^: Chi square

POL-β; DNA polymerase beta, DCIS; Ductal Carcinoma *in Situ.* METABRIC: Molecular Taxonomy of Breast Cancer International Consortium. HER2; Human epidermal growth factor receptor 2.

**Supplementary material and method S1**

**Tissue Microarray Construction (TMA)**

**Samples preparation**

FFPE donor blocks of pure DCIS and DCIS coexisting with the invasive disease were retrieved from the NHS archive (Nottingham Health Science Biobank). 4µm full-face tissue sections from the retrieved blocks were stained with Haematoxylin and Eosin (H&E), reviewed by light microscope in order to confirm and select the suitable area of DCIS lesion avoiding the necrotic areas. On the reviewed glass slides, selective lesions were marked and matching with FFPE blocks. Digital scanning was achieved by Panoramic digital scanner (250 Flash III, 3 DHISTECH, then the digital images viewed by using Panoramic Digital Viewer v. 1.15.4 (3DHISTECH) digital marking was performed.

**Recipient blocks**

Well mounted cassettes with melted regular paraffin were cooled on ice in order to harden the paraffin. The borders of the paraffin blocks were trimmed by using microtome to ensure the flat surface of the blocks with the recommended thickness of the recipient blocks (5-10mm).

**TMA construction**

Donor and recipient blocks were loaded into full automated TMA Grand Master® (3D HISTECH®, Budapest, Hungary). Cores with 1 mm diameter were taken from the donor blocks. In order to ensure the efficient construction steps, 4µm tissue sections were sectioned from the ready recipient blocks and stained with H&E for quality control purposes.

**Supplementary material and method S 2**

The assessment of the expression of POLβ protein in DCIS by immunohistochemistry (IHC) was conducted using the Novocastra Novolink polymer detection system (Leica, Newcastle, UK) following manufacturer’s guidelines. 4µm TMA and full face tissue sections were cut from FFPE blocks and heated for 10 minutes at 60 °C. Slides were loaded into autostainer (Leica autostainer XL), for dewaxing / rehydrating sections (xylene 2x5 min, industrial methylated spirit (IMS) 3x2 min, 2 min distiller water, 5 min tap water wash). Slides were treated with heat-assisted antigen retrieval methods. Citrate Buffer pH 6.0 was used as antigen retrieval with microwave 1000W heated for 20 min. Peroxidase block and protein block (Novolink Kit) were applied for 5 min to each slide to minimise background staining and prevent non-specific binding. Afterwards, TBS-tween 20 (Tris Buffered Saline pH 7.6 with tween 20, 1%) was used to rinse slides for 5 min which was repeated 3 times after each step. Subsequently, samples were incubated for 30 min in room temperature with the POL*β* primary antibody optimally diluted at 1:500 in Leica antibody diluent followed by TBS tween and were rinsed 3 times for 5 min each. This was followed by post-primary and polymer (Novolink Kit) were applied for 3 0 min each, rinsed with TBS tween 20 3x 5 min and repeated after each step. Then, chromogenic DAB solution (3, 30-Diaminobenzidine tetrahydrochloride, Novolink DAB substrate buffer) was applied for 5 min. Haematoxylin was used as counterstain for 6 min (Novolink Haematoxylin). A positive control of testis sections were included, while a negative control was achieved by excluding the primary antibody. Slides were loaded into the autostainer machine, in order to dehydrated and cleared (IMS 3x2 min; xylene 2x2 min; and 5 min tap water wash). Finally, the slides were cover-slipped using DPX mounting and left overnight to dry.
